# Dysfunctional β-cell autophagy induces β-cell stress and enhances islet immunogenicity

**DOI:** 10.3389/fimmu.2025.1504583

**Published:** 2025-01-29

**Authors:** Matthew C. Austin, Charanya Muralidharan, Saptarshi Roy, Justin J. Crowder, Jon D. Piganelli, Amelia K. Linnemann

**Affiliations:** ^1^ Department of Biochemistry & Molecular Biology, Indiana University School of Medicine, Indianapolis, IN, United States; ^2^ Department of Medicine, Indiana University School of Medicine, Indianapolis, IN, United States; ^3^ Department of Pediatrics, Indiana University School of Medicine, Indianapolis, IN, United States; ^4^ Indiana Center for Diabetes and Metabolic Diseases, Indiana University School of Medicine, Indianapolis, IN, United States

**Keywords:** type 1 diabetes, autophagy, β-cell stress, islet immunogenicity, HLA-I expression

## Abstract

**Background:**

Type 1 Diabetes (T1D) is caused by a combination of genetic and environmental factors that trigger autoimmune-mediated destruction of pancreatic β-cells. Defects in β-cell stress response pathways such as autophagy may play an important role in activating and/or exacerbating the immune response in disease development. Previously, we discovered that β-cell autophagy is impaired prior to the onset of T1D, implicating this pathway in T1D pathogenesis.

**Aims:**

To assess the role of autophagy in β-cell health and survival, and whether defects in autophagy render islets more immunogenic.

**Methods:**

We knocked out the critical autophagy enzyme, ATG7, in the β-cells of mice (ATG7^Δβ-cell^) then monitored blood glucose, performed glucose tolerance tests, and evaluated bulk islet mRNA and protein. We also assessed MHC-I expression and presence of CD45+ immune cells in ATG7^Δβ-cell^ islets and evaluated how impaired autophagy affects EndoC-βH1 HLA-I expression under basal and IFNα stimulated conditions. Lastly, we co-cultured ATG7^Δβ-cell^ islet cells with diabetogenic BDC2.5 helper T cells and evaluated T cell activation.

**Results:**

We found that all ATG7^Δβ-cell^ mice developed diabetes between 11-15 weeks of age. Gene ontology analysis revealed a significant upregulation of pathways involved in inflammatory processes, response to ER stress, and the ER-associated degradation pathway. Interestingly, we also observed upregulation of proteins involved in MHC-I presentation, suggesting that defective β-cell autophagy may alter the immunopeptidome, or antigen repertoire, and enhance β-cell immune visibility. In support of this hypothesis, we observed increased MHC-I expression and CD45+ immune cells in ATG7^Δβ-cell^ islets. We also demonstrate that HLA-I is upregulated in EndoC β-cells when autophagic degradation is inhibited. This effect was observed under both basal and IFNα stimulated conditions. Conversely, a stimulator of lysosome acidification/function, C381, decreased HLA-I expression. Lastly, we showed that in the presence of islet cells with defective autophagy, there is enhanced BDC2.5 T cell activation.

**Conclusions:**

Our findings demonstrate that β-cell autophagy is critical to cell survival/function. Defective β-cell autophagy induces ER stress, alters pathways of antigen production, and enhances MHC-I/HLA-I presentation to surveilling immune cells. Overall, our results suggest that defects in autophagy make β-cells more susceptible to immune attack and destruction.

## Introduction

Type 1 Diabetes (T1D) is one of the most common chronic conditions in children, and its incidence is increasing (1). From 2017 to 2020, the number of people diagnosed with T1D in the United States increased nearly 30% (2). T1D develops following autoimmune-mediated destruction of pancreatic β-cells (3–6). Genetic risk factors in combination with a hypothesized environmental triggering event (such as a viral infection) leads to immune activation in disease pathogenesis. A critical gap in knowledge exists in our understanding of T1D development concerning whether β-cells are innocent bystanders, or contribute to their own immune targeting and destruction (7–9). Recent evidence suggests that β-cell stress pathways play an important role in activating and/or exacerbating the immune response in disease development (10–12). This includes the discovery that islet reactive CD8+ T cells are present at comparable numbers and activity level, in the circulation of T1D patients compared to healthy controls (13). The authors described this surprising finding as a state of “benign” autoimmunity in healthy controls. They reasoned that activation of these T cells may rely on enhanced β-cell susceptibility to islet inflammation in individuals with T1D (13).

One stress response pathway implicated in the development of T1D is β-cell macroautophagy, often simply referred to as autophagy (14). The autophagy pathway scavenges and degrades cellular proteins and organelles, including damaged structures, under conditions of stress (15–17). Previously, we discovered that autophagy is impaired in the islets of the nonobese diabetic (NOD) mouse model of T1D as well as in islets from human donors with T1D (18). Importantly, this pathway is impaired prior to the onset of T1D, implicating this pathway in T1D pathogenesis. In support of a role for defective autophagy in T1D pathogenesis, some non-HLA T1D susceptibility genes are involved in autophagy, including cathepsin H (CTSH) a lysosomal protease (19). Another link between defective autophagy and β-cell autoimmunity is the formation of hybrid insulin peptides (HIPs), proinsulin peptide fragments fused with other insulin granule peptide fragments. These hybrid peptides are neoantigens that are recognized by autoreactive T cells in T1D (20, 21). Importantly, evidence suggests that autophagy pathways are an important source of HIP formation (22–24).

β-cell autophagy could also play an important role in cell susceptibility to environmental triggering events leading to T1D development. One leading hypothesis for an environmental triggering event in T1D is viral infection (25). In support of this theory, enterovirus virus antigen was detected in pancreatic specimen from T1D patients at 10x the rate of controls (26). Furthermore β-cells infected with enterovirus exhibit impaired autophagic flux (27). This impaired flux may lead to long-standing low levels of infection, non-lytic spread of the virus, and contribute to triggering autoimmunity (27). Autophagy can also play a protective role against infection, by slowing viral endocytosis and preventing excessive activation of interferon pathways in infected cells (28). Indeed, in proteomics performed in an autophagy deficient cell line, viral sensing proteins in the RIG-1 pathway mediating interferon response accumulate (29). This suggests autophagy plays an important role in modulating the interferon response during infection.

Under conditions of impaired autophagy, the balance between a protective vs. deleterious interferon response could be tipped and lead to β-cell autoreactivity. There is good evidence that high levels of interferon are deleterious to the β-cell, leading to autoimmune attack. Interferon-α (IFNα) therapy for both hepatitis C infection and hairy cell leukemia has been associated with T1D development (30–32). In addition, an elevated type-I interferon signature (including IFNα) is observed in pancreatic islets from individuals with recent onset T1D, as well as in the peripheral blood of individuals at risk for developing T1D (33, 34). Though there are evident links between viral infection, impaired autophagy, and T1D, it has not been determined whether impaired autophagy in isolation can recapitulate events linked to viral infection in T1D development. These events include induction of endoplasmic reticulum (ER) stress, signatures of interferon response, neoantigen formation, and β-cell death (35).

Based on these observations, we set out to characterize the mechanisms by which alterations in β-cell autophagy might contribute to T1D pathogenesis. We hypothesized that defective β-cell autophagy may alter the β-cell-immune cell interface thus playing a role in activating and/or exacerbating the immune response in T1D development. To test our hypothesis, we employed a mouse model of impaired β-cell autophagy alongside pharmacological modulators of autophagy in mouse islets and human β-cells. We demonstrate here that disruption of autophagy leads to changes indicative of β-cell stress coinciding with altered MHC-I presentation and enhanced islet immunogenicity.

## Methods

### EndoC-βH1 cell culture

The EndoC-βH1 cell line present in this study was obtained from Human Cell Design. EndoC-βH1 cells were cultured as previously described (36). Briefly, cells were cultured on plates coated with matrigel (100 μg/mL, Corning, 356237) plus fibronectin (2 μg/mL), in DMEM (5.6 mM glucose) supplemented with 2% BSA, nicotinamide (10 mM), transferrin (5.5 μg/mL), sodium selenite (6.7 ng/mL), 2-mercaptoethanol (50 μM), and penicillin/streptomycin (100 U/mL, 100 μg/mL respectively). Passages were performed weekly, and cells were tested periodically for mycoplasma contamination (Applied Biological Materials, G238).

### ATG7^Δβ-cell^ mouse line

To generate the ATG7^Δβ-cell^ C57BL6/J mice, we employed a Cre-Lox system where constitutive *Ins1^Cre^* mice were crossed with *Atg7^fl/fl^* mice. *Atg7^fl/fl^* mice (B6.Cg-Atg7<tm1Tchi>) were obtained with permission from the donating investigator from RIKEN (RRID: IMSR_RBRC02759). The *Ins1^Cre^* mice (B6(Cg)-Ins1tm1.1(Cre)Thor/J) were obtained from Jackson Labs (RRID: IMSR_JAX:026801).

### Mouse phenotyping and tissue harvest

Random fed blood glucose levels were assessed by tail prick twice weekly starting at 6 weeks of age using an AlphaTrak2 glucometer (Zoetis). For Kaplan-Meier curves, diabetes was defined as consecutive readings of blood glucose >250 mg/dL (>13.9 mmol/L) on separate days. At 8 weeks of age, a glucose tolerance test was performed following an overnight fast. Each mouse was injected intraperitoneally with 2 mg glucose per gram body weight and blood glucose was measured just prior to injection, and at indicated time points up to 2 hours following glucose injections. For experiments utilizing isolated islets, ATG7^Δβ-cell^ and littermate control mice were sacrificed via cervical dislocation, and islets were harvested by the Indiana University School of Medicine (IUSM) Islet and Physiology Core as previously described (37). Mice were similarly sacrificed and pancreata harvested for immunofluorescence analysis. For mouse tissue harvest, islet isolations and pancreata harvest were performed several weeks prior to diabetes development (7-10 weeks of age) from ATG7^Δβ-cell^ and littermate control mice. Experimental mice were housed in a temperature-controlled facility with a 12-hour light, 12-hour dark cycle and had free access to food and water. All experiments were approved by the IUSM Institutional Animal Care and Use Committee.

### Western blot analysis

Following islet isolation, islets were washed and incubated in lysis buffer containing 50 mM Tris-HCl (pH 8.0), 1 mM EDTA (pH 8.0), 150 mM NaCl, 5% glycerol, 0.05% sodium deoxycholate, 0.2% sarkosyl, 10 mM NaF, 0.1% IGEPAL, 0.1% SDS, 1mM DTT, 2 mM MgCl_2_, 0.05% w/v benzonase, and 1x Protease Inhibitor Cocktail (Fisher Scientific, NC0818307). Lysis incubation occurred for 20 minutes on ice and samples were vortexed every 5 minutes to aid lysis. Samples were then passed through a 22-gauge needle 10x and stored at -80 °C until blotting was performed. Sample protein concentration was determined using Pierce™ Rapid Gold BCA Protein Assay Kit (Thermo Fisher Scientific, A53226) per manufacturer’s guidelines. Samples were mixed with 4x loading buffer (LI-COR, D00908-01) with 1:10 β-mercaptoethanol (50 μL total sample volume), boiled at 95 °C for 10 minutes, and run on pre-cast gels (BioRad, 4561094). Protein was then transferred to activated PVDF membranes for analysis using anti-ATG7 (Abcam, ab133528, 1:1,000) and anti-β-actin (CST, 3700S, 1:1,000) antibodies at 4 °C overnight followed by incubation with donkey anti-mouse IRDye 680RD (LI-COR Biosciences, 926-68072, 1:10,000) and donkey anti-rabbit IRDye 800CW (LI-COR Biosciences, 926-32213, 1:10,000) for 1 hour at room temperature. Blots were imaged on a LI-COR Odyssey Imager.

### ATG7^Δβ-cell^ islet transcriptomic analysis

Following islet isolation, islets were rested overnight prior to RNA extraction using RNAeasy Plus Mini Kit (Qiagen, 74134) per manufacturer’s guidelines. Quality of RNA was assessed at the IUSM Center for Medical Genomics using Agilent 2100 Bioanalyzer. Dual-indexed cDNA libraries were prepped using SMART-seq ultra-low input RNA kit (Takara Bio) and sequenced using NovaSeq6000 (Illumina). The sequenced libraries were mapped to mouse genome UCSC mm10 and differential expression was calculated at the IUSM Center for Medical Genomics and Bioinformatics core. ATG7^Δβ-cell^ mRNA transcript levels were plotted as fold change (FC) compared to control levels. Genes with a FC greater than 1.5 or less than -1.5 were considered up or downregulated respectively. A p-value threshold of ≤0.05 was also used for the selection of both up and downregulated genes. Differentially regulated genes were subjected to Gene Ontology and functional analysis using DAVID (https://davidbioinformatics.nih.gov/), a free online functional annotation tool (38, 39). The plots were generated using GraphPad Prism.

### ATG7^Δβ-cell^ islet proteomic analysis

In addition, protein was isolated from ATG7^Δβ-cell^ and littermate control islets and prepared for mass spectrometry analysis. Islets were washed in PBS, spun down, decanted, pellet flash frozen in liquid nitrogen, and samples submitted to the IUSM Center for Proteome Analysis for further processing. Briefly, samples were resuspended in 8 M urea, reduced in 100 mM Tris (pH 8.5), alkylated, enzymatically digested (Promega trypsin/LysC mix, 1:70 ratio), and desalted (Waters SepPak). Samples were subsequently TMT labeled, mixed, dried, and desalted. The prepared sample was then loaded and separated on an ultra-high performance liquid chromatographer (Easynano LC1200) using a C18 Aurora 25cm nanocolumn coupled to an Eclipse Orbitrap mass spectrometer (Thermo Fisher). Following spectra generation, peptides were identified using Proteome Discoverer 2.5 searching the Mus musculus protein database, with common contaminants included. Proteins with a FC greater than 1 or less than -1 were considered up or downregulated respectively. A p-value threshold of ≤0.05 was also used for the selection of both up and downregulated proteins. Gene Ontology and functional analysis was performed using DAVID and plotted by GraphPad Prism.

### Immunofluorescence analysis

Following tissue harvest, pancreata were embedded in optimal cutting temperature compound and frozen on dry ice prior to slicing tissue sections for immunofluorescence staining. Frozen 8 µm pancreatic sections were cut at -20°C (Leica CM1860 Cryostat) and adhered to poly-L-lysine coated slides. Tissue sections were then fixed in acetone for 10 min at RT, washed in PBS, incubated in protein blocking solution (Abcam, AB64226), followed by incubation in antibody diluent (Abcam, ab64211) with 40 μg/mL AffiniPure Fab Fragment (Jackson ImmunoResearch, 115-007-003) at 4 °C overnight. The next day slides were washed in PBS, PBS 0.1% Tween 20, then PBS prior to overnight incubation at 4 °C in antibody diluent containing primary antibodies at the following dilutions: anti-insulin 1:2 (Dako, a0564), anti-MHC-I 1:150 (Biolegend, 114602), and anti-CD45 1:50 (BD Biosciences, 550539). Slides were similarly washed as before prior to incubation in the dark at room temperature for 1 hour in antibody diluent containing secondary antibodies and nuclear stain at the following dilutions: Alexa Fluor 488 goat anti-guinea pig 1:500 (Invitrogen, A11073), Alexa Fluor 594 goat anti-mouse 1:500 (Invitrogen, A11032), Alexa Fluor 647 goat anti-rat 1:500 (Invitrogen, A21247), and DAPI 1:2,000. Slides were then washed and mounted with EverBrite Mounting Medium (Biotium, 23001) and imaged on a Leica Stellaris 5 confocal microscope using a 63x/1.40 oil objective.

Image analysis was then performed using ImageJ in a semi-automated fashion. Briefly, insulin positive area was used to determine a region of interest (ROI) wherein mean islet fluorescent intensity for MHC-I was measured. Within this ROI, CD45+ nuclei were also counted, as well as total nuclei, to determine the percentage of CD45+ cells within each islet. Background fluorescence was subtracted using the mean fluorescent intensity value of islets from serial sections treated with 2^nd^ antibodies only as a control. Data was plotted using GraphPad Prism.

### Flow cytometry

EndoC-βH1 cells were treated overnight with 100 nM Bafilomycin A1 (Millipore Sigma, 508409), 100 μM C381 (MedChemExpress, HY-100347A), or vehicle control, DMSO. Subsequent experiments included pre-treatment for 24 hours with 2,000 IU/mL recombinant IFNα 2 protein (Novus Biologicals, NBP2-26551) followed by washout with PBS, replacement with EndoC-βH1 culture media, culture for 72 hours prior to overnight treatment with 100 nM Bafilomycin A1, 100 μM C381, or DMSO. Following overnight treatment, cells were washed, removed from the culture plate using 0.05% trypsin, and spun down at 500x g for 5 minutes. Cells were resuspended and washed in FACS buffer containing 1% bovine serum albumin in 1x dPBS, 0.1% sodium azide, and 0.02% w/v EDTA. Cells were then incubated in FACS buffer containing HLA-ABC Class I primary conjugated Alexa Fluor 488 (clone: W6/32) for 30 minutes at 4 °C (BD Biosciences 567851), washed, and fixed in 1% paraformaldehyde in PBS on ice. Cells were subsequently washed, resuspended in FACS buffer, and analyzed using an Attune Nxt acoustic flow cytometer.

### Dispersed islet cell and BDC 2.5 splenocyte co-culture

Spleens were harvested from 8-week-old NOD.Cg-Tg(TcraBDC2.5, Strain-004460) homozygous mice, and splenocytes were isolated as previously described (40). Briefly, spleens from BDC2.5 mice were processed by mashing through 70 µm strainer (Corning, CLS352350) and then treated with red blood cell lysis buffer (Sigma Aldrich, R7757) for 2 minutes, followed by washing twice with complete DMEM media. For *in vitro* experiments, 500,000 splenocytes cells were plated per well in a 96 round bottom plate (Corning, 3799) and cultured in complete DMEM media. Concurrently, islets were isolated from ATG7^Δβ-cell^ and Cre+ control mice. Islets from 3-6 ATG7^Δβ-cell^ and 3-6 Cre+ control mice were pooled, and half the control islets were treated with 100 nM Bafilomycin A1 overnight prior to co-culture. To set up the co-culture, islets were spun down, washed with PBS, resuspended in Accutase (Fisher Scientific, NC9839010), and incubated in a water bath for 10-15 minutes at 37 °C. Islets were pipetted vigorously every few minutes and an aliquot was checked under a microscope to confirm islet dissociation into single cells. Islet cells were then counted using a hemocytometer to add the same amount of islet cells per condition (3,600 and 1,800 cells) to splenocytes previously plated. As a positive control, BDC2.5 splenocyte cells were stimulated with 0.05 µM of BDC2.5 peptide (2.5HIP: RGG-LQTLALWSRMD-GGR) and 2.5 µg/ml Concanavalin A (Sigma, C2272) respectively. Each condition was performed in triplicate. At 24, 48, and 72 hours, samples were spun down and the supernatant was collected to measure IFNγ secretion by ELISA.

### Cytokine measurement by ELISA

Culture supernatant from different time points were measured for mouse IFNγ cytokine level. 96 well high affinity flat bottom plate (Corning, 3361) were coated with 25 ng/mL monoclonal rat anti-murine IFNγ (BD Pharmaceutical, 551216) by diluting in carbonate buffer (pH 9.6) overnight at 4 °C. For detection, biotin rat anti-mouse IFNγ (Clone XMG1-2, BD Pharmingen, 554410) and Streptavidin-HRP (Invitrogen, 434323) were used according to manufacturer’s protocol. The plate was read on Spectramax M5 microplate reader (Molecular Devices) at OD 450 nm and data analyzed using SoftMax Pro version 7.0.2. Data summarizes results from two independent experiments. Each condition was performed in triplicate and 9 Cre+ control and 9 ATG7^Δβ-cell^ mice were used between experiments.

### Statistical analysis

Statistical analyses were performed using GraphPad Prism v10.2. Pairwise comparisons between two groups were conducted using an unpaired two-tailed Student’s t-test. For experiments where HLA-I expression was normalized to control levels for each replicate, a one sample t-test was performed, with a hypothetical population mean equal to 1. One-way ANOVA was used to compare the means from multiple treatment groups, with Dunnett’s *post hoc* test adjusted for multiple comparisons. Histogram plots are represented as mean or median ± standard deviation, and dot plots are represented as mean ± standard error of the mean. A p-value <0.05 was considered statistically significant.

## Results

### β-cell Atg7 deletion causes diabetes in mice

**Figure 1:**
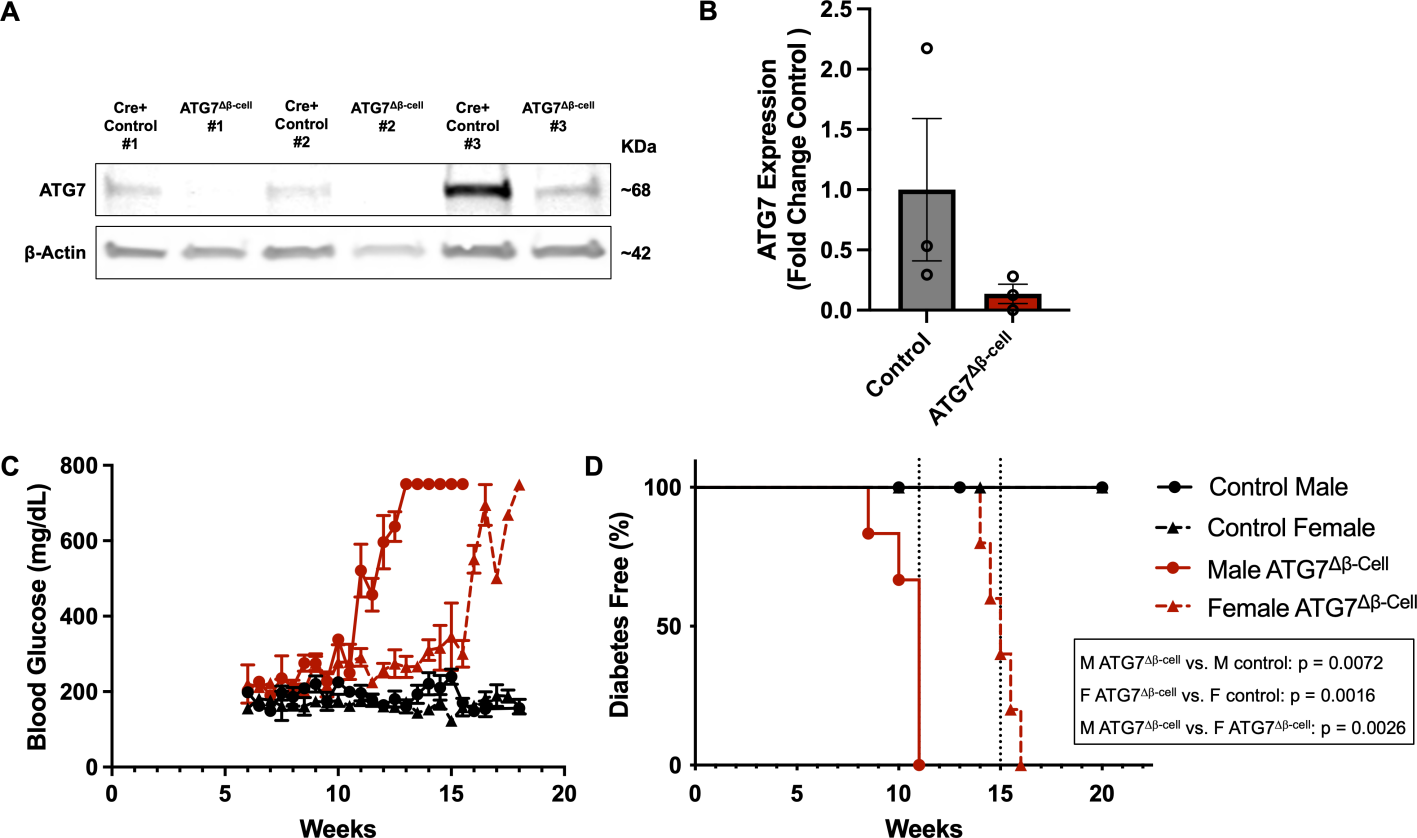
Loss of β-cell ATG7 leads to hyperglycemia and diabetes development. **(A)** Representative western blot images showing ATG7 levels in ATG7^Δβ-cell^ and littermate control mouse islets with quantification relative to β-actin shown in **(B)** as mean ± SD. **(C)** Random fed blood glucose measurements performed twice weekly beginning at 6 weeks of age in ATG7^Δβ-cell^ (n=5 female, n=6 male) and littermate control mice (n=7 female, n=5 male), plotted as mean ± SEM. **(D)** Kaplan-Meier diabetes free curve for male (n=6) and female (n=5) ATG7^Δβ-cell^ mice compared to littermate control male (n=5) and female (n=7) mice. Vertical dashed line represents median age of diabetes development in male (11 weeks) and female (15 weeks) ATG7^Δβ-cell^ mice. Diabetes was defined as two consecutive blood glucose measurements >250 mg/dL on consecutive days.

To better understand the role of autophagy in β-cell function and survival, we selectively knocked out the critical autophagy enzyme, autophagy related gene 7 (ATG7), in the β-cells of mice (ATG7^Δβ-cell^ mice, see **Figures 1A, B**). ATG7 knockout in a variety of target tissues has elucidated the critical role of autophagy in various cell types (41). The ATG7 enzyme is essential for the expansion and maturation of autophagosomes, thus rendering ATG7 knockout cells severely autophagy deficient (42). In humans, recessive variants causing severe loss of the ATG7 enzyme, lead to neurodevelopmental disorders affecting the brain and muscle, with more subtle evidence of endocrine hypofunction also present in these individuals (42). Previous mouse models of β-cell autophagy deficiency have shown evidence of impaired glucose homeostasis and glucose stimulated insulin secretion, as well as reduced insulin content and β-cell mass (43, 44). Here, we conducted bi-weekly random fed blood glucose measurements, confirming previous findings of early onset progressive hyperglycemia leading to overt diabetes development in ATG7^Δβ-cell^ mice (**Figures 1C, D**) (44). There was also evidence of impaired glucose tolerance as early as 8 weeks in male mice, but not yet in females, as indicated by glucose tolerance challenge (**Supplementary Figures 1A, B**). However, we found that females develop similar levels of hyperglycemia and diabetes with a delayed onset compared to males, which was not previously appreciated (**Figures 1C, D**). All ATG7^Δβ-cell^ mice developed diabetes, with male ATG7^Δβ-cell^ mice having a median age of 11 weeks at diabetes onset and female ATG7^Δβ-cell^ mice a median age of 15 weeks at diabetes onset (**Figure 1D**).

### ATG7^Δβ-cell^ islet transcriptomics/proteomics reveals enhanced β-cell stress and potential susceptibility to triggering events

**Figure 2:**
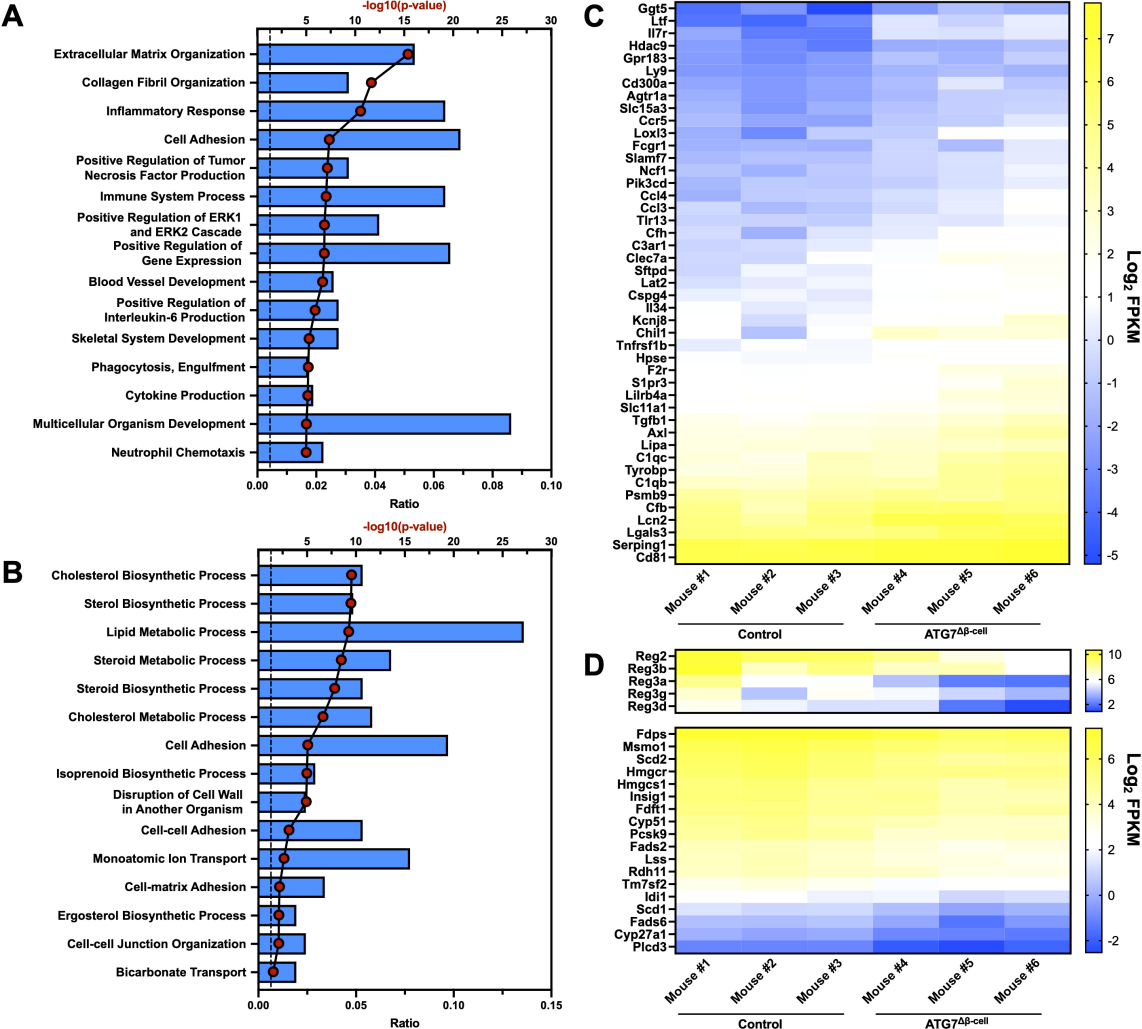
Islet transcriptional changes induced by ATG7^Δβ-cell^ knockout. Top 15 transcriptionally **(A)** upregulated or **(B)** downregulated biological process pathways identified by DAVID analysis. The -log_10_(Benjamini adjusted p-value) for each category are illustrated by red dots and the ratio of pathway hits to total hits are represented by blue histograms. The vertical dashed line represents a Benjamini adjusted p-value cutoff of <0.05. Heat maps showing relative abundance of genes associated with **(C)** upregulated inflammatory/immune response pathways or **(D)** downregulated *Reg* genes (top) and genes associated with cholesterol/lipid metabolism (bottom). The genes selected from these pathways for heat map display were chosen by using a p-value cutoff of <0.01. mRNA sequencing reads were normalized to fragments per kilobase transcript, per million mapped reads (FPKM).

Next, we aimed to understand the islet biological process pathways altered by dysfunctional β-cell autophagy in the ATG7^Δβ-cell^ model. To do this, we evaluated bulk islet mRNA and protein levels by RNA-sequencing and mass spectrometry, respectively. Islets were isolated several weeks prior to diabetes development to assess pathways that are altered leading to overt disease. At the transcript level, we observed an upregulation of transcripts involved in extracellular matrix and collagen fibril organization pathways (**Figure 2A**). This includes upregulation of metalloproteinases, which are involved in extracellular matrix remodeling. This remodeling could be indicative of, and necessary for, immune cell recruitment in ATG7^Δβ-cell^ islets, as has been observed in T1D pathology (45, 46). The upregulation of these pathways could also indicate a fibrotic response that has been observed with loss of ATG7 in other tissues (47).

In addition, we found a significant upregulation of transcripts involved in the inflammatory response and immune system processes (**Figures 2A, C**). Upregulation of these pathways suggested potential enhanced immune cell recruitment to ATG7^Δβ-cell^ islets, as both upregulation of chemotactic factors and receptors was observed (**Figure 2C**). We also observed upregulation of transcripts involved in tumor necrosis factor (TNF) and interleukin-6 (IL-6) production, cytokines that have been linked to early events in T1D development including immune cell recruitment and activation (**Figure 2A**) (48–50). This suggests that in the absence of a hyperactive immune system, due to absence of NOD genetic risk factors on the C57BL6/J background, defective autophagy and concurrent ER stress might be sufficient to promote key features of inflammation in diabetes development. This is supported by a recent study that showed that pharmacological inhibition of the maladaptive ER stress response can delay onset of autoimmune diabetes in NOD mice (51).

We also identified genes in several key pathways that were downregulated at the transcript level, including those associated with cholesterol and lipid biosynthesis and metabolism (**Figures 2B, D**). Evidence from the literature suggests ER stress negatively regulates cholesterol export and metabolism, which could explain the downregulation of transcripts involved in these pathways (52, 53). There was also downregulation of the *Regenerating (Reg)* family of genes that are associated with β-cell proliferation, protection from apoptosis, and protection from cytokine induced damage (**Figure 2D**) (54–56). This suggests that these processes are potentially impaired in ATG7^Δβ-cell^ islets.

**Figure 3:**
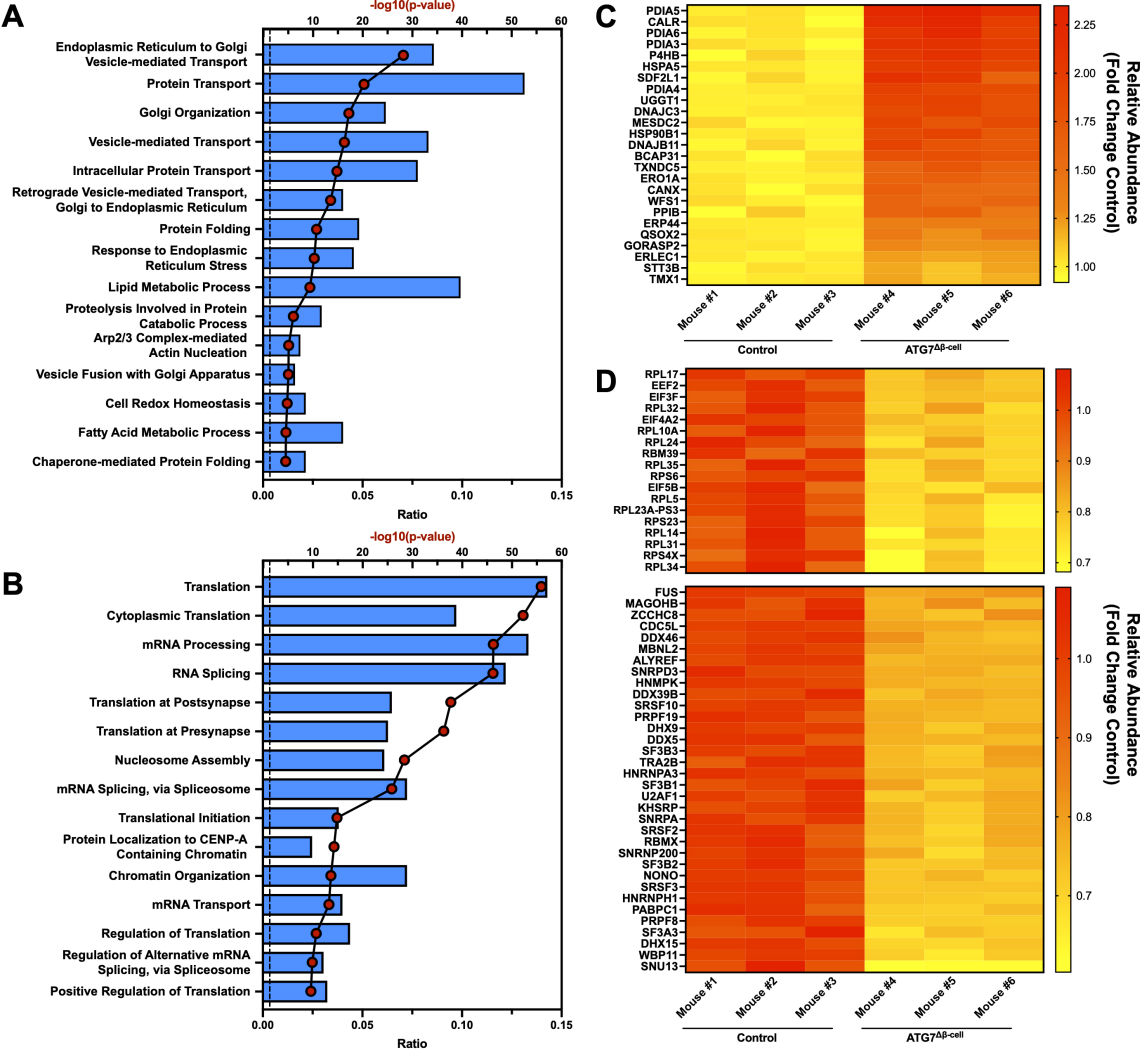
Islet proteomic changes induced by ATG7^Δβ-cell^ knockout. Top 15 proteomic **(A)** upregulated or **(B)** downregulated biological process pathways identified by DAVID analysis. The -log_10_(Benjamini adjusted p-value) for each category are illustrated by red dots and the ratio of pathway hits to total hits are represented by blue histograms. The vertical dashed line represents a Benjamini adjusted p-value cutoff <0.05. Heat maps showing relative abundance of top proteins associated with **(C)** upregulated protein folding, ER stress, and ERAD pathways or **(D)** downregulated proteins associated with translation (top) and mRNA splicing/processing (bottom). The proteins selected from these pathways for heat map display were chosen by using a p-value cutoff of <0.01. Knockout mouse islet protein levels were normalized to littermate control islet levels.

It is widely observed that transcript changes do not always directly correlate to protein changes, as was recently shown very broadly in human islets from numerous donors (57). At the protein level, we saw an upregulation of proteins involved in response to ER stress, with a concomitant increase in chaperone proteins involved in protein folding as well as proteins involved in the ER associated degradation (ERAD) pathway (**Figures 3A, C**). Downregulated proteins were associated with pathways involved in protein translation, mRNA splicing, and mRNA processing, which are consistent with the ER stress induced by ATG7^Δβ-cell^ knockout (**Figures 3B, D**). Several of the most strongly upregulated proteins were unsurprisingly associated with the autophagy pathway that are accumulating under conditions of impaired autophagic flux. This includes receptors that target proteins for autophagic degradation and aid autophagic vesicle maturation. Upregulation of compensatory protein degradation pathways to deal with accumulating cellular waste, typically targeted for autophagic degradation, included enhanced proteasome-mediated proteolysis and protein secretory pathways (**Figure 3A**) (57). We also found several of the upregulated ER stress response proteins are also involved in antigen production and MHC-I complex assembly. This included chaperones that stabilize the complex before peptide loading (ERp57/PDIA3, CNX), help facilitate peptide binding as well as recruit other members of the complex (ERp57/PDIA3, CRT), and transport it to the plasma membrane (BCAP31) (58). Many subunits of the proteasome, which processes proteins for MHC-I presentation, are also upregulated. A shunting of proteins typically targeted for autophagic degradation to alternate protein degradation pathways could have broad implications for the antigen repertoire presented by the β-cell to surveilling immune cells.

Next, we looked at the intersection of genes differentially regulated at both the transcript and protein level. Because of the limited number of genes identified from this intersection, the pathway analysis was not separated into upregulated and downregulated genes. Many of the pathways previously discussed appeared in our analysis, including response to ER stress, protein folding, and ERAD pathways (**Supplementary Figure 2A**). To determine whether these pathways were primarily up or down regulated, we plotted gene transcript fold change (FC) vs. protein FC (**Supplementary Figure 2B**). We observed that genes associated with the response to ER stress and protein folding pathways were almost exclusively downregulated at the transcript level and upregulated at the protein level when autophagy is defective (**Supplementary Figure 2B**). This suggests that reduced degradation of these proteins, and subsequent feedback inhibition on transcription, limits further accumulation of proteins in these pathways. Similarly, receptors that mediate selective autophagic degradation of the ER during ER stress, such as CCPG1, are upregulated at both the transcript and protein level (59). Finally, DMBT1 and ZIP8—among the most strongly downregulated factors—are specifically linked to immune defense and negatively regulate proinflammatory response (60, 61).

### ATG7^Δβ-cell^ loss enhances islet MHC-I expression and immune cell recruitment

**Figure 4:**
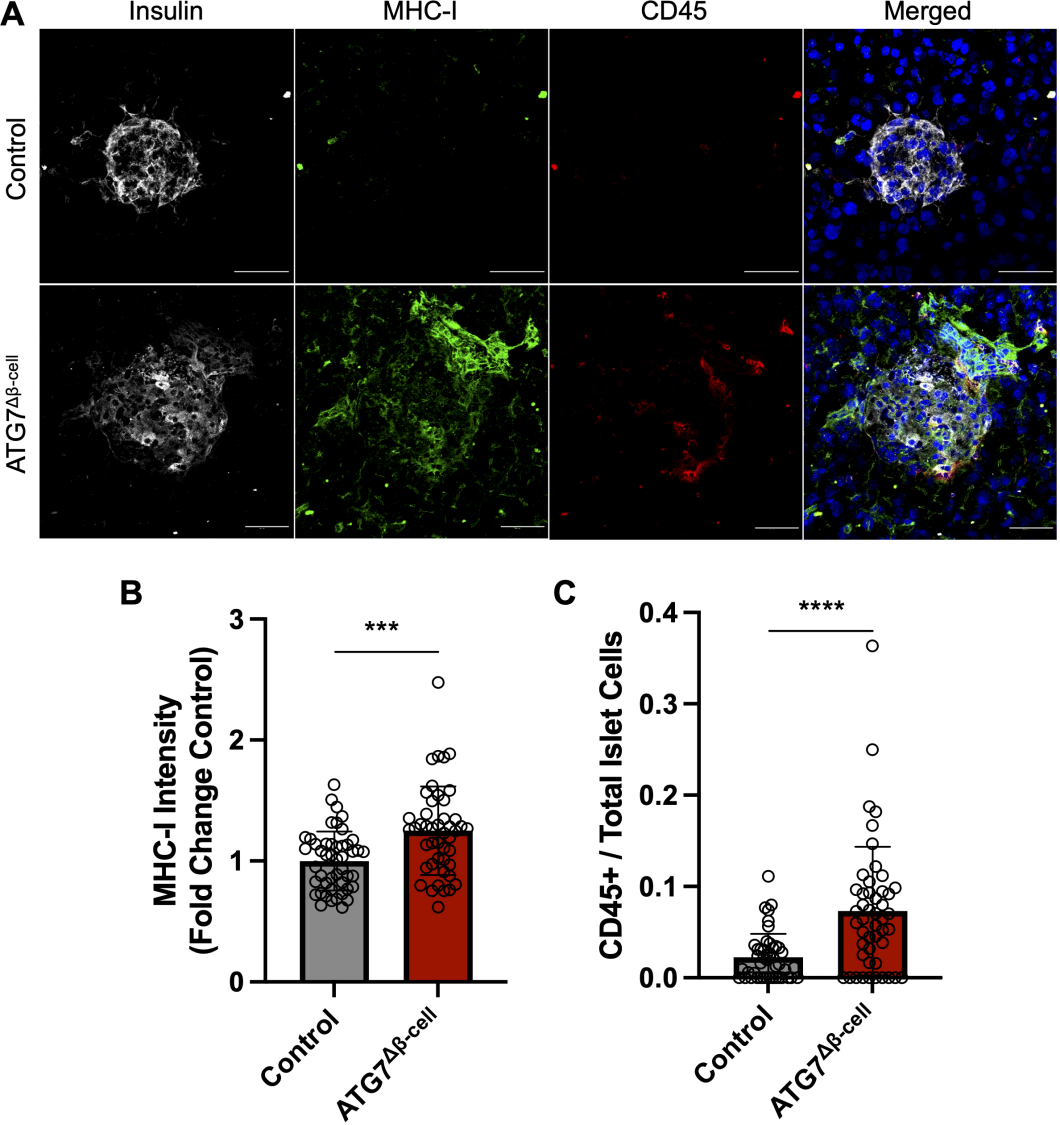
ATG7^Δβ-cell^ islets display enhanced MHC-I expression and CD45+ immune cell recruitment. **(A)** Representative images of fresh frozen pancreatic sections stained with antibodies against insulin (gray), MHC-I (green), CD45 (red), and DAPI nuclear stain (blue). Scale Bar = 50 μm. **(B)** Quantification of islet MHC-I mean fluorescence intensity normalized to littermate control levels. Each circle represents an islet (n=4 mice per genotype, 11-15 islets quantified per mouse). *** p≤ 0.001 by unpaired t-test, data plotted as mean ± SD. **(C)** Quantification of CD45+ cells given as a ratio compared to total number of islet cells. Each circle represents an islet (n=4 mice per genotype, 10-15 islets quantified per mouse). **** p≤ 0.0001 by unpaired t-test, data plotted as mean ± SD.

Due to alterations of proteins involved in antigen production and MHC-I complex assembly, we next asked whether MHC-I expression was altered in ATG7^Δβ-cell^ islets. Therefore, fresh frozen pancreas tissue sections were assessed for MHC-I islet expression. We observed a significant increase in mean MHC-I intensity in ATG7^Δβ-cell^ islet area compared to control islets (p = 0.0002, **Figures 4A, B**). This result is consistent with other models of dysfunctional autophagy in immune cells that also demonstrated increased MHC-I expression (62, 63). In humans, there is also evidence of enhanced HLA Class I (HLA-I, human homolog of MHC-I) expression in individuals who harbor recessive variants causing severe loss of the ATG7 enzyme (42). Therefore, impaired autophagy could be a mechanism that contributes to HLA-I islet hyperexpression in T1D, a well-known feature of disease development (64–68).

In addition to potentially enhancing β-cell visibility to immune cells via MHC-I upregulation, we assessed whether the ATG7^Δβ-cell^ islets showed altered immune cell presence within the islet area. We immunostained for CD45+ cells in the pancreata of ATG7^Δβ-cell^ and control islets and observed a significant increase in the number of CD45+ immune cells in and around ATG7^Δβ-cell^ islets compared to littermate controls (p < 0.001, **Figures 4A, C**). This discovery coincides with our transcriptomic data showing upregulated inflammatory response and cytokine production in ATG7^Δβ-cell^ islets.

### Pharmacological modulation of autophagy alters HLA-I expression under basal and IFNα stimulated conditions

**Figure 5:**
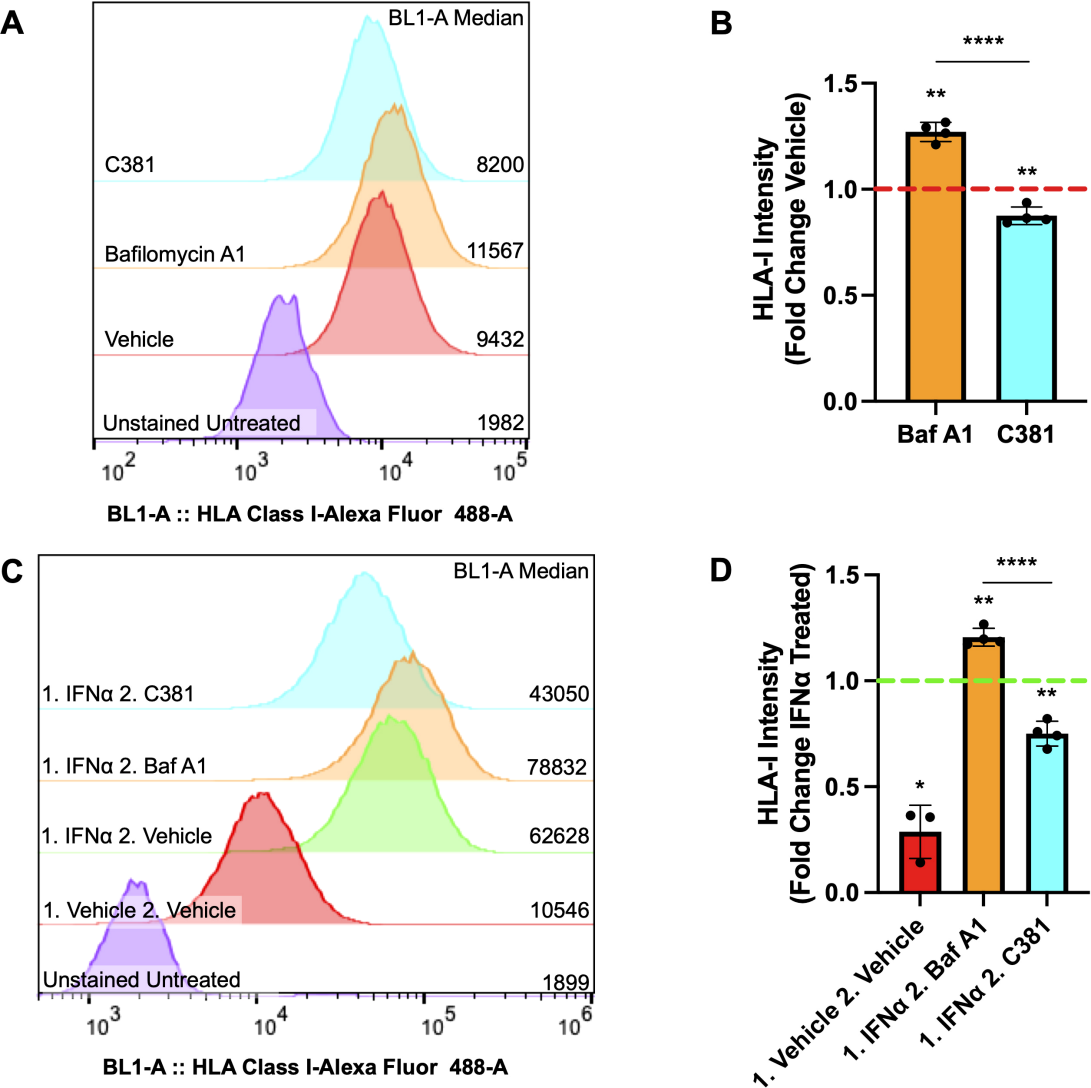
Autophagy modulating compounds alter HLA-I expression under basal and IFNα stimulated conditions. **(A)** Representative sample distribution plot showing HLA-I expression measured by flow cytometry in EndoC-βH1 cells treated for 16-18 hours with vehicle control (red), 100 nM Bafilomycin A1 (orange), or 100 μM C381 (cyan). Raw median fluorescent intensity from each curve is displayed on the right side of the graph. **(B)** HLA-I median fluorescence intensity ± SD plotted as fold change relative to vehicle control, which is represented by the red dashed line. **p≤ 0.01, ****p≤ 0.0001 by one sample t-test (vehicle mean = 1) or unpaired t-test to compare treatment means (n= 4 per treatment condition). **(C)** Representative sample distribution plot showing HLA-I expression measured by flow cytometry in EndoC-βH1 cells treated for 24 hours with IFNα or vehicle control (red), followed by 16-18 hours with vehicle control (green), 100 nM Bafilomycin A1 (orange), or 100 μM C381 (cyan) after washout. Raw median fluorescence intensity from each curve is displayed on the right side of the graph. **(D)** HLA-I median fluorescence intensity ± SD plotted as fold change relative to IFNα treated, followed by vehicle treated control, which is represented by the green dashed line. *p≤ 0.05, **p≤ 0.01, ****p≤ 0.0001 by one sample t-test (control mean = 1) or unpaired t-test to compare treatment means (n= 3-4 per treatment condition).

To determine if our ATG7^Δβ-cell^ mouse model observations would replicate in human cells, we also assessed whether pharmacological modulation of autophagy in EndoC-βH1 cells would alter HLA-I expression. EndoC-βH1 cells were treated overnight with the lysosome acidification inhibitor Bafilomycin A1 (Baf A1), to inhibit the last stage of autophagic degradation, or vehicle control. Alternatively, cells were treated with a novel small molecule, C381, known to stimulate lysosome acidification/function (formerly known as SRI-011381) (69). After treatment, surface HLA-I expression was analyzed using flow cytometry. We found that treatment with the inhibitor Baf A1 enhanced human β-cell HLA-I expression, whereas stimulating lysosome function with C381 treatment had the opposite effect and reduced HLA-I expression (p = 0.0012 and p = 0.0094 respectively, **Figures 5A, B**). Previously, our lab demonstrated evidence for impaired lysosome function in β-cells both in the setting of T1D and in autoantibody positive individuals, who have not yet developed T1D (18). Thus, impaired β-cell autophagy could be a mechanism that contributes to the HLA-I hyperexpression that is thought to be induced primarily by proinflammatory cytokines (64, 70).

Following this result, we wanted to determine whether autophagy modulation following IFNα induced HLA-I hyperexpression would alter β-cell HLA-I surface expression. To test this, EndoC-βH1 cells were treated for 24 hours with IFNα, the treatment was washed out, and then followed with overnight treatment with the autophagy inhibitor Baf A1, stimulator C381, or vehicle control. The washout period was determined by when the literature indicates β-cell HLA-I expression induced by IFNα treatment should begin declining to return to baseline levels (71). Following treatment, surface HLA-I expression was again assessed by flow cytometry. Our results demonstrate that Baf A1 treatment maintains the hyperexpression of HLA-I induced by IFNα above vehicle treatment following washout, whereas stimulating lysosome function by C381 treatment accelerates the disappearance of HLA-I following IFNα treatment (p = 0.0023 and p = 0.0034 respectively, **Figures 5C, D**). This result, in combination with observations from the literature, suggests that impaired autophagy could exacerbate the effects of IFNα on β-cells tipping the balance from a protective to a maladaptive interferon response (29, 70).

### Impaired β-cell autophagy increases islet cell immunogenicity

**Figure 6:**
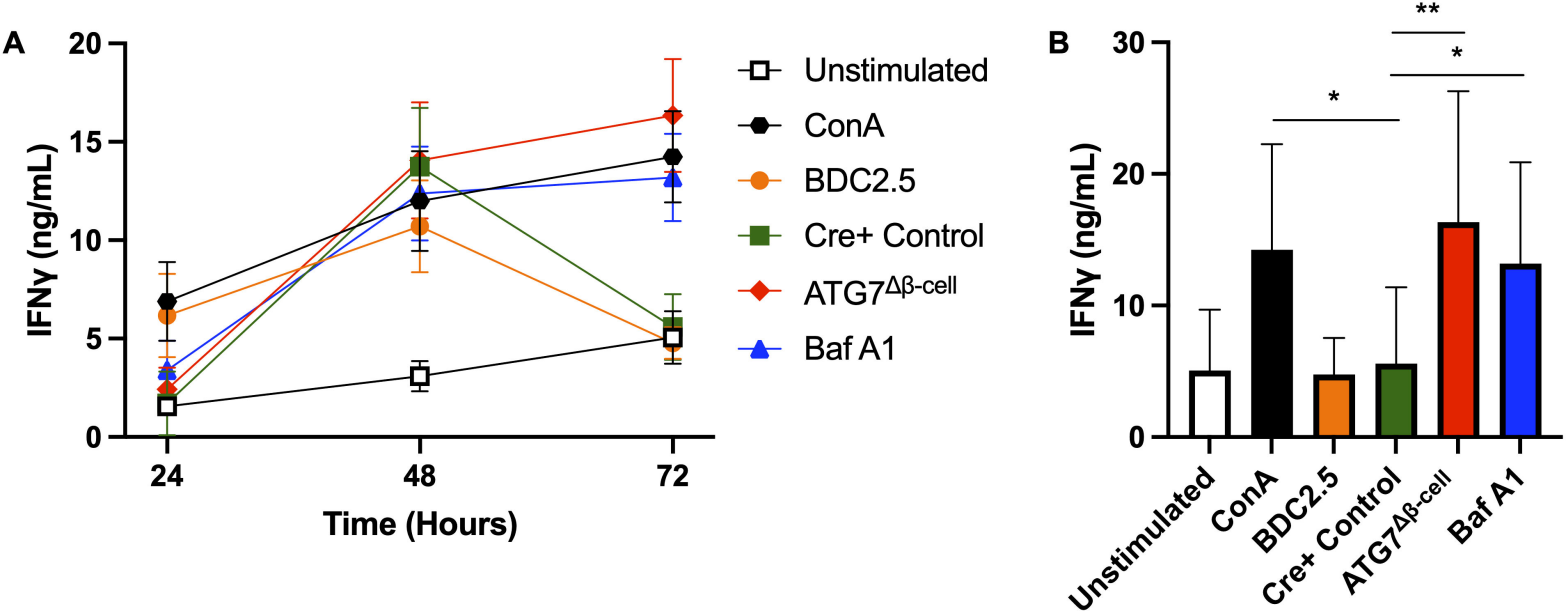
Loss of islet and β-cell autophagy enhances BDC2.5 T cell IFNγ production in co-culture. **(A)** Splenocytes were co-cultured for 24-72 hours with 3,600 dispersed islet cells from ATG7^Δβ-cell^ (red diamonds), Cre+ control (green squares), or Bafilomycin A1 treated (100 nM) Cre+ control islets (blue triangles), and IFNγ production was measured by ELISA from co-culture supernatant. Splenocytes were stimulated with 0.05 µM BDC2.5 peptide (orange circles) or 2.5 µg/ml Concanavalin A (black hexagons) as a positive control, or unstimulated as a negative control (white square). Data plotted as mean ± SEM. **(B)** Histogram plot of IFNγ supernatant levels at the 72-hour time point. *p≤ 0.05, **p≤ 0.01 by one-way ANOVA, with data plotted as mean ± SD.

Taken together, our results suggested that impaired β-cell autophagy may make β-cells more immunogenic and susceptible to immune attack and destruction. Impaired autophagy could increase β-cell visibility to CD8+ T cells through enhanced HLA-I expression, and may also allow for the buildup and/or release of misfolded and improperly modified peptides, including neoantigens. These peptides can then be taken up by antigen presenting cells for presentation on HLA-I and HLA-II complexes to prime effector cells in T1D pathogenesis. To begin testing this hypothesis, we co-cultured dispersed ATG7^Δβ-cell^ or wild type islet cells, pretreated with Baf A1 or vehicle control, with splenocytes from BDC2.5 T cell receptor transgenic mice that contain diabetogenic CD4+ T cells. Following 24-72 hours of co-culture, T cell stimulation was assessed by collecting the cell supernatant and measuring Interferon-γ (IFNγ) production by ELISA. We found that in the presence of dispersed cells from ATG7^Δβ-cell^ islets, there is sustained elevation of IFNγ production at 72 hours compared to littermate control islet cells (p = 0.0014, **Figures 6A, B**). Furthermore, we found that in the presence of wild type islet cells pretreated with Baf A1, there is a similar sustained elevation of IFNγ production at 72 hours compared to vehicle treated control islet cells (p = 0.0373, **Figures 6A, B**). We also obtained strikingly similar results when we performed the experiment with a dilution of islet cells (**Supplementary Figures 3A, B**). The IFNγ levels induced by islet cells with defective autophagy were comparable to levels induced by a positive control treated with Concanavalin A, a potent control of polyclonal activation of all T cells. This suggests that dysfunctional β-cell autophagy could make β-cells more immunogenic and susceptible to immune attack and destruction, as indicated by the enhanced BDC2.5 T cell activation. One could hypothesize that the delay in autophagic clearance of damaged or mis-folded proteins may lead to their accumulation and subsequent recognition by the β-cell reactive T cells, similar to previous observations in the NOD model (72).

## Discussion

Our previous work demonstrated that autophagic flux is impaired in the setting of T1D (18). This was shown by reduced co-localization of autophagosome and lysosome markers in pancreata of both NOD mice and human organ donors with T1D, in combination with the absence of autophagosome marker accumulation upon pathway inhibition in the NOD mouse model (18). Importantly, evidence of impaired β-cell autophagy and lysosome function was also found in the pancreata of human donors at risk for developing T1D (auto-antibody positive), prior to T1D onset (18). This led us to propose that impaired β-cell autophagy plays a role in T1D development.

Our findings in this study helped to characterize the role of defective autophagy more thoroughly in β-cell dysfunction/death and to evaluate more directly its potential role in altering the β-cell-immune cell interface. We demonstrated that β-cell autophagy is critical to cell survival/function, as dysfunctional β-cell autophagy led to diabetes development in the ATG7^Δβ-cell^ model. Furthermore, ATG7^Δβ-cell^ islet transcriptomics and proteomics revealed that defective β-cell autophagy induces an ER stress and inflammatory response, as well as alters pathways of antigen production/presentation. This corresponded with enhanced immune cell recruitment to ATG7^Δβ-cell^ islets, and enhanced MHC-I/HLA-I surface expression under conditions of impaired autophagy. Conversely, stimulating autophagy led to reduced HLA-I expression. Lastly, we demonstrated that impaired islet and β-cell autophagy enhances BDC2.5 T cell activation in co-culture. Overall, our results suggest that defects in autophagy make β-cells more susceptible to immune attack and destruction.

As mentioned, we showed that loss of the critical autophagy enzyme, ATG7, leads to the development of diabetes, and at a sex dependent rate. The similar incidence, but delayed onset of diabetes in female mice, was not previously appreciated in the ATG7^Δβ-cell^ model. One potential explanation for the delayed onset in female mice, could be recent evidence suggesting that human female β-cells are more resilient to ER stress mediated damage than age matched male controls (73). This includes a more robust unfolded protein response as well as maintaining higher levels of insulin secretion under conditions of ER stress (73). Unlike many autoimmune diseases that show significantly higher prevalence in women, T1D appears to occur at slightly higher rates in men (74, 75). It seems plausible that this could be due, at least in part, to the differential β-cell ER stress response in women compared to men.

To better understand the mechanisms of β-cell loss in ATG7^Δβ-cell^ mice, we performed transcriptomic and proteomic analysis on ATG7^Δβ-cell^ and control islets. Given the observed upregulation of proteins involved in the ER stress response, it seems likely that the ER stress induced by ATG7^Δβ-cell^ loss plays a primary role in β-cell death in this model. Previous studies have primarily focused on apoptosis as the means of ER stress induced β-cell death (76). However, a recent report demonstrated that an alternate more inflammatory form of cell death, called necroptosis, is induced by ER stress in β-cells (76). Follow up experiments will determine how β-cell death is induced in this model—through apoptosis, necroptosis, or another route. Interestingly, our data showed an upregulation of transcripts involved in TNF production, and it has been demonstrated that TNF-induced β-cell death is regulated by mediators of necroptosis (77).

In addition, we also observed upregulation of transcripts involved in IL-6 production, which has been shown to both play a role in T1D development as well as promote protective β-cell responses under certain contexts (48, 50, 78–80). Along with islet resident immune cells, β-cells themselves have been shown to be an important source of inflammatory cytokines and chemokines, including CXCL10 and IL-6 (81–83). These findings, combined with published observations that secretory autophagy is a pathway for proinflammatory cytokine secretion, provides the possibility that β-cell autophagosome/lysosome exocytosis could act as a chemoattractant to immune cells (84–86). Pathways of secretory autophagy and lysosomal exocytosis have been shown to be stimulated under conditions of impaired autophagy (87, 88). Thus, future experiments will assess the role of defective autophagy on β-cell cytokine production and immune cell chemotaxis.

Along with the recruitment of immune cells, dysfunctional β-cell autophagy may contribute to enhancing β-cell visibility to surveilling immune cells. In professional antigen presenting cells, it has been demonstrated that loss of autophagy (including loss of ATG7) leads to enhanced MHC-I expression (62, 63). Our data revealed that loss of ATG7 in β-cells also enhances β-cell MHC-I expression. Furthermore, we demonstrated that pharmacological inhibition of autophagy via Baf A1 treatment enhances surface level expression of HLA-I in human β-cells. In contrast, treating with a stimulator of lysosomal acidification and function, C381, led to decreased HLA-I expression. These changes in HLA-I expression were observed both under basal conditions and following IFNα induced HLA-I hyperexpression. Previous studies have attributed elevated MHC-I/HLA-I expression under conditions of impaired autophagy to reduced endocytosis of the complex (63). The study did not detect transcriptional changes, alterations to complex formation or shuttling rate to the plasma membrane (63). Additionally, our ATG7^Δβ-cell^ proteomics data did not show changes to proteins specific to the peptide loading complex associated with MHC-I, such as tapasin. This further suggests that the increased MHC-I/HLA-I expression we observed is due to reduced endocytosis or enhanced recycling of the complex, instead of enhanced formation/shuttling of new complex.

HLA-I islet hyperexpression is a well-defined characteristic of T1D, with studies indicating this occurs prior to insulitis development (89, 90). The primary driver of HLA-I hyperexpression in T1D are cytokines including IFNα. Additionally, the type-I interferon response is thought to be associated with events that trigger autoimmunity (33, 64, 91, 92). However, it is plausible that defective β-cell autophagy could be a mechanism contributing to increased HLA-I expression on β-cells that is seen in T1D. Our data supports the possibility that following an inflammatory insult such as a viral infection, and subsequent induction of islet HLA-I expression, defective β-cell autophagy helps maintain enhanced HLA-I expression when cytokine levels begin to fall as the infection is cleared. More broadly, our results support evidence in the literature that indicates impaired β-cell autophagy may exacerbate the effects of IFNα on β-cells, including ER stress, HLA-I overexpression, and apoptosis (70). This could tip the scales from a protective interferon-mediated response to an overactive response leading to β-cell attack and destruction.

In addition to enhancing β-cell visibility to immune cells via HLA-I upregulation, dysfunctional autophagy may also enhance alternate routes of antigen production leading to neoantigen formation. It is well documented that a significant fraction of HLA-I molecules recycle to the plasma membrane following clathrin independent internalization and subsequent endosome sorting (93). Intriguingly, this allows for the possibility of an alternate route of peptide loading on recycled HLA-I, and a potential source of neoantigen presentation (12). In the autophagy pathway, autophagosomes can fuse with endosomes (forming amphisomes) that will be targeted for lysosomal degradation or recycled to the cell surface. Under conditions of impaired autophagy, it is observed that cells will undergo secretory autophagy and lysosomal exocytosis to prevent waste build up and maintain cellular homeostasis (87, 88). This may thus serve as a route for improperly modified or digested peptides to be processed and presented by antigen presenting cells. Furthermore, proteins that are preferentially degraded through the autophagy pathway, such as proinsulin, could have altered expression in the β-cell antigen repertoire when there is a shift to primarily proteasomal degradation (94). Our observation of enhanced expression of proteins involved in proteasome-mediated proteolysis and protein secretory pathways in the ATG7^Δβ-cell^ islets, supports the possibility of increasing alternate routes of peptide loading, protein/peptide secretion, and potential changes to the β-cell immunopeptidome.

In support of our hypothesis that defective autophagy may make β-cells more visible and/or antigenic to immune cells, we demonstrated enhanced activation of BDC2.5 T cells in the presence of islets cell with impaired autophagy. The ligand for diabetogenic BDC2.5 T cells is 2.5HIP that consists of an insulin C-peptide fragment fused with the N-terminus of a chromogranin A peptide fragment, WE14 (21). A recent report found that ER stress induces increased formation of 6.9HIPs in primary NOD islet cells (24). Thus, conditions of defective β-cell autophagy and concomitant induction of ER stress may favor enhanced 2.5HIP formation to drive the enhanced T cell activation that we observed. In addition to discovering enhanced HIP production under conditions of ER stress, this study also localized 6.9HIPs to crinosome and dense core granule (DCG) sub-cellular compartments in β-cells by electron microscopy (24). Another study demonstrated that crinophagy is strongly suppressed in ATG7 knockout β-cells (95). These findings, in conjunction with our previous findings that crinophagy is reduced in β-cells from auto-antibody positive individuals, suggest that increased levels of HIPs may escape intracellular degradation and be co-secreted with DCGs under conditions of impaired autophagy (18). This would allow for enhanced uptake and subsequent presentation of HIPs on antigen presenting cells to activate diabetogenic T cells.

While defective β-cell autophagy has been demonstrated in the setting of T1D and in individuals at risk for developing T1D, a causative role of this pathway in disease development has not previously been established. One major obstacle has been separating the specific effects of autophagy modulation in β-cells vs. immune cells. To highlight this complexity, systemic administration of drugs that either stimulate or inhibit autophagy have both been shown to reduce diabetes incidence in NOD mice, with unclear answers as to the specific mechanism that elicits the effect (96, 97).

It is also important to highlight the limitations of the ATG7^Δβ-cell^ model. While this mouse model is useful to isolate the effects of dysfunctional autophagy on β-cells and the β-cell-immune cell dialogue, it does not fully recapitulate the complex genetic and environmental factors leading to T1D development. Additionally, the ATG7^Δβ-cell^ model more broadly and potently inhibits autophagy than the defects likely present in human T1D pathogenesis. Despite these limitations, this model provides valuable insights into the mechanisms by which changes in β-cell autophagy might contribute to T1D pathogenesis. For instance, we provide evidence that both genetic and chemical inhibition of islet and β-cell autophagy renders the β-cells more immunogenic than their respective controls. We also identified several potential pathways whereby defective β-cell autophagy may enhance β-cell antigenicity, and thus play a role in T1D development. These include promoting production of inflammatory cytokines, enhancing deleterious IFNα-mediated effects, and enhancing β-cell visibility via HLA-I upregulation.

Conversely, our results also suggest that stimulating β-cell autophagy to reverse these effects could have therapeutic utility in the context of T1D. Recently, there has been increased interest in drugs that target β-cells to alter T1D progression (98). Approved drugs such as verapamil (L-type calcium channel blocker) have been repurposed to test their efficacy in T1D. Clinical trial results suggest that verapamil promotes β-cell function, delays β-cell loss, and reduces exogenous insulin requirements in people with recent onset T1D (98, 99). Another compound, tauroursodeoxycholic acid, has been shown to reduce β-cell ER stress in T1D mouse models, and is currently being tested in clinical trials (98, 100). Furthermore, glucagon-like peptide-1 receptor agonists (GLP-1RAs) are another promising, adjunctive therapy targeting the β-cell in T1D (101). GLP-1RAs reduced HbA1c, body weight, and insulin dose requirements in large scale T1D clinical trials, with these effects attributed to residual β-cell function (102–104). The mechanism conferring these effects likely includes enhancing β-cell proliferation, survival, and function, including by alleviating ER stress (105–107). However, it is worth noting that GLP1R agonism can also stimulate β-cell autophagy, suggesting that stimulation of this pathway more selectively in the β-cell might also yield beneficial effects (108, 109). It remains to be tested whether pharmacological modification of β-cell autophagy can impact T1D development. However, recent efforts to package and deliver therapeutics selectively to the β-cell will soon allow for β-cell targeted therapies to assess the utility of the autophagy pathway in treating autoimmune diabetes (110). In this context, our work suggests that future studies targeting β-cell autophagy may be a viable approach to prevent or delay autoimmune diabetes.

## Data Availability

The mRNA sequencing data presented in the study have been deposited in the Gene Expression Omnibus repository, accession number GSE287583. The mass spectrometry proteomics data have been deposited to the ProteomeXchange Consortium via the PRIDE partner repository with the dataset identifier PXD060032 (111).

## Supplementary material

Supplementary Figure 1 Male ATG7^Δβ-cell^ show signs of impaired glucose tolerance at 8 weeks. **(A)** Intraperitoneal glucose tolerance test performed in male (left) and female (right) 8-week-old ATG7^Δβ-cell^, ATG7^fl/-^, Cre+ and littermate control mice and **(B)** area under the curve (AUC) was plotted. *p≤ 0.05 by unpaired t-test at each time point, with data plotted as mean ± SEM and mean ± SD respectively. AUC p-values determined by one-way ANOVA.

Supplementary Figure 2 Intersection of transcriptional and proteomic changes induced by ATG7^Δβ-cell^ knockout. **(A)** Differentially regulated biological process pathways at both the transcript and protein level identified by DAVID analysis. The -log_10_(Benjamini adjusted p-value) for each category are illustrated by red dots and the ratio of pathway hits to total hits are represented by blue histograms. The vertical dashed line represents a Benjamini adjusted p-value cutoff <0.05. **(B)** Genes that had a p-value < 0.05 at the transcript and protein level were plotted to visualize transcript log_2_ fold change vs. protein log_2_ fold change. Genes/proteins associated with the response to ER stress (red circles) and protein folding (blue triangles) predominantly clustered in the bottom right quadrant. Select genes upregulated and downregulated at both levels are labeled.

Supplementary Figure 3 Loss of islet and β-cell autophagy enhances BDC2.5 T cell IFNγ production in co-culture after islet cell dilution. **(A)** Splenocytes were co-cultured for 24-72 hours with 1,800 dispersed islet cells from ATG7^Δβ-cell^ (red diamonds), Cre+ control (green squares), or Bafilomycin A1 treated (100 nM) Cre+ control islets (blue triangles), and IFNγ production was measured by ELISA from co-culture supernatant. Splenocytes were stimulated with 0.05 µM BDC2.5 peptide (orange circles) or 2.5 µg/ml Concanavalin A (black hexagons) as a positive control, or unstimulated as a negative control (white square). Data plotted as mean ± SEM. **(B)** Histogram plot of IFNγ supernatant levels at the 72-hour time point. *p≤ 0.05, **p≤ 0.01 by one-way ANOVA, with data plotted as mean ± SD.
